# Persistent socioeconomic and racial and ethnic disparities in pathogen burden in the United States, 1999–2014

**DOI:** 10.1017/S0950268819001894

**Published:** 2019-11-11

**Authors:** R. C. Stebbins, G. A. Noppert, A. E. Aiello, E. Cordoba, J. B. Ward, L. Feinstein

**Affiliations:** 1 Department of Epidemiology, University of North Carolina – Chapel Hill, Chapel Hill, NC, USA; 2 Carolina Population Center, University of North Carolina – Chapel Hill, Chapel Hill, NC, USA; 3 Social and Scientific Systems, Durham, NC, USA

**Keywords:** Herpesviruses, human papilloma virus, pathogen burden, race/ethnicity, socioeconomic status

## Abstract

The disproportionate burden of prevalent, persistent pathogens among disadvantaged groups may contribute to socioeconomic and racial/ethnic disparities in long-term health. We assessed if the social patterning of pathogen burden changed over 16 years in a U.S.-representative sample. Data came from 17 660 National Health and Nutrition Examination Survey participants. Pathogen burden was quantified by summing the number of positive serologies for cytomegalovirus, herpes simplex virus-1, HSV-2, human papillomavirus and *Toxoplasma gondii* and dividing by the number of pathogens tested, giving a percent-seropositive for each participant. We examined sex- and age-adjusted mean pathogen burdens from 1999–2014, stratified by race/ethnicity and SES (poverty-to-income ratio (PIR); educational attainment). Those with a PIR < 1.3 had a mean pathogen burden 1.4–1.8 times those with a PIR > 3.5, with no change over time. Educational disparities were even greater and showed some evidence of increasing over time, with the mean pathogen burden among those with less than a high school education approximately twice that of those who completed more than high school. Non-Hispanic Black, Mexican American and other Hispanic participants had a mean pathogen burden 1.3–1.9 times non-Hispanic Whites. We demonstrate that socioeconomic and racial/ethnic disparities in pathogen burden have persisted across 16 years, with little evidence that the gap is closing.

## Background

Socioeconomic and racial/ethnic disparities in health have been reported in the literature for decades, with those in lower socioeconomic groups and members of racial and ethnic minorities often experiencing higher proportions of and more severe adverse health events. These disparities exist across a range of health outcomes, including infectious diseases [1, 2], cognition and Alzheimer's disease [3], cancers [4], reproductive outcomes [5], cardiovascular health outcomes [6], and injuries [7]. While some of this heterogeneity in the distribution of health and disease by sociodemographic factors can be explained by differential access to care or health behaviours [8], a growing body of research points to the potential role of prevalent, persistent pathogens as an important mechanism through which social factors lead to differences in long-term health [9–12].

Persistent pathogens are pathogens that establish latent infections in the body and are never cleared but are maintained in a latent state by the immune system. Examples of these infections include herpesviruses, such as herpes simplex virus-1 (HSV-1), cytomegalovirus (CMV) and varicella zoster virus; *Toxoplasma gondii* (*T. gondii*); and many other viruses, bacteria and parasites. Often these infections cause no clinical symptoms, with some key exceptions, including congenital CMV infection. Due to their lack of acute health effects, many of these pathogens have been under-studied, historically, in both epidemiologic and biomedical research.

More recently, research has suggested that these latent infections may have long-term direct health consequences [13–16]. For example, researchers have shown that CMV partially mediates the relationship between socioeconomic status (SES) and mortality [13] and that inflammatory cytokines mediate the relationship between CMV seropositivity and mortality [16]. Many of these latent infections are thought to affect health through similar immunologic pathways, including: (1) direct tissue destruction via localised inflammation, whereby pathogens found in various organs elicit a local immune response in which the immune cells attack the local tissue [17]; (2) the triggered release of pro-inflammatory cytokines (innate immune response), which increases the number of circulating inflammatory markers [18] or (3) molecular mimicry, a process through which antibodies targeted against such pathogens may cross-react with and attack host tissues expressing proteins homologous to those contained by the pathogen [19]. The overlap in the mechanisms through which each latent infection impacts the body suggests that infection with multiple pathogens may have a cumulative health impact above and beyond infection with a single pathogen, and points to total pathogen burden (i.e. the cumulative number of pathogens that an individual is infected with) as a potentially important indicator of health status. Indeed, high levels of total pathogen burden are now an established risk factor for atherosclerosis and cardiovascular disease [20, 21].

Socially disadvantaged groups (e.g. low SES, racial/ethnic minorities) have a higher prevalence of latent infections, higher immunoglobulin G (IgG) antibodies to those infections and higher levels of total pathogen burden. For example, low SES has been associated with a higher prevalence of CMV, Epstein–Barr virus (EBV) and HSV-1 [12, 22]. Low SES has also been associated with a greater total number of persistent infections across the life course in the United States (U.S.), from childhood [10] through adulthood [11, 23]. Similarly, consistent disparities by racial and ethnic categories in the U.S. have also been seen with individual infections [22, 24, 25] and burden [11], with non-Hispanic Whites having the lowest prevalence and burden compared to racial/ethnic minorities such as non-Hispanic Blacks and Hispanic populations. Importantly, these disparities remain after adjustment for SES [11, 22, 24, 25].

While there is strong evidence for socioeconomic and racial/ethnic disparities in pathogen burden [10–12, 22–26], whether and how these patterns have changed over time is currently unknown. In this paper, we use data from a nationally representative U.S. sample to quantify the magnitude of racial/ethnic and socioeconomic disparities in pathogen burden and assess how this disparity has changed over a 16-year period.

## Methods

### Study population

The data for the present study come from the 1999–2014 National Health and Nutrition Examination Surveys (NHANES), a population-based survey that uses a multistage stratified probability sample designed to provide nationally representative estimates of the civilian noninstitutionalised U.S. population. The 16 years of data were collected and pooled across eight survey waves. Each wave consisted of an independent, cross-sectional, U.S. representative survey. Full details on the NHANES study design and response rates have been published previously [27–29].

All adults aged 18–49 years old who participated in the laboratory component of the survey [30] and who had valid test results were eligible for inclusion in the present analysis (*n* = 24 269). The laboratory subset is a random subset of the NHANES population and, using appropriate survey weighting procedures designed by NHANES, is representative of the civilian noninstitutionalised U.S. population at this age group [30]. We excluded observations with missing data on infections, income, education, and/or race/ethnicity for a final sample size of 17 660 across the 16-year study period (Figure S1). The NHANES study protocols were approved by the Research Ethics Board of the National Center for Health Statistics, CDC.

### Measures

#### Pathogen burden

The outcome for this analysis was pathogen burden, a composite measure of seropositivity to several pathogens, including: CMV, HSV-1, HSV-2, human papillomavirus (HPV) and *T. gondii.* The pathogens chosen were based on their ability to establish chronic infections in participants, their availability in NHANES and considerations about treatment availability and vaccination effects. For example, Hepatitis B was excluded due to the high prevalence of vaccination to that virus. EBV, though a herpesvirus, was also excluded due to only being measured in children ages 6 to 19 years. HPV, however, was included as the vaccine was only approved in 2006 for women and 2009 for men and recommended for ages 11–12, as well as 13–26 if not already vaccinated. Therefore, the earlier years of HPV data are not affected, and the later years would only have a small proportion of the NHANES populations affected by the vaccine: women ages 18–26 in 2006 or men ages 21–26 in 2009 who received the vaccine and aged into the eligible population by 2014. The specific pathogens included in the mean pathogen burden score calculations varied over the study period and their prevalences are shown in Table 1. Details on the laboratory testing procedures and information on which HPV strains were tested can be found in the CDC NHANES Laboratory Data documentation [30].

**Table 1.** Descriptive statistics of 1999–2014 NHANES population by study wave, *n* = 17 660

	1999–2000	2001–2002	2003–2004	2005–2006	2007–2008	2009–2010	2011–2012	2013–2014
*N*	1775	2349	2097	2332	2327	2484	2190	2112
HSV-1 (% seropositive)	62.5	62.1	59.6	58.1	59.6	56.1	58.0	52.4
HSV-2 (% seropositive)	21.2	20.9	19.3	19.6	18.5	17.7	18.4	14.3
CMV (% seropositive)	55.8	54.7	52.2	–	–	–	–	–
HPV (% seropositive)	–	–	25.0	36.7	28.9	32.7	6.5	6.8
*T. gondii* (% seropositive)	–	11.7	13.4	–	–	11.0	9.0	9.2

For each participant, we quantified pathogen burden by summing the number of pathogens for which the participant was seropositive. To account for differences in the total number of pathogens assessed over time, we standardised the pathogen burden measure by dividing the total number of pathogens for each participant by the number of available pathogens in that year and multiplied by 100, resulting in a percentage ranging from 0–100. We also conducted a sensitivity analysis, replicating our results with a pathogen burden measure that only represented HSV-1 and HSV-2 burden, which were measured across all years.

#### Sociodemographics

There were three exposures for this analysis: poverty-to-income ratio (PIR), educational attainment, and racial and ethnic categorization.

The PIR was calculated by dividing the total family income by the annual poverty threshold as determined by the U.S. Census Bureau, based on the household size. A PIR < 1 indicates that family income was below the poverty threshold. Four categories were created for household income: low (PIR < 1.30), low-middle (PIR ⩾ 1.30 to ⩽1.85), middle (PIR > 1.85 to ⩽3.50), and high (PIR > 3.50). This categorization scheme is based on the U.S. Department of Agriculture's Supplemental Nutrition Assistance Program (SNAP) and Special Supplemental Nutrition Program for Women, Infants and Children (WIC) programs' income eligibility cut-points for food assistance through SNAP (PIR ⩽ 1.30) or WIC (PIR ⩽ 1.85) as recommended in the NHANES Analytic and Reporting Guidelines [31]. The highest income group (PIR > 3.50) was used as the reference category.

We categorised educational attainment into three groups based on highest degree achieved: less than a high school diploma, high school diploma and/or some college, and college degree and/or graduate education, based on recommended cut points in the NHANES guidelines [31]. The highest education category was used as the reference group for analysis.

We utilised the following racial and ethnic categories provided by NHANES and available at all waves: Mexican American, Other Hispanic, Non-Hispanic White, Non-Hispanic Black, and Other Race/Multi-Racial. For all comparisons, Non-Hispanic White served as the reference group. The Other Race/Multi-Racial category was excluded as an exposure since it represents a collapsed group of many different races and ethnicities, including several categories of Asian American, Native Americans, and those who identify as multi-racial. As such, we cannot provide meaningful interpretation of this category, though if more granular measures are available in future waves, they would be worth exploring.

#### Covariates

All analyses were adjusted for age (continuous) and biological sex (dichotomous). Each sociodemographic indicator (PIR, educational attainment, and race/ethnicity) was assessed in a separate analysis that did not include adjustment for the other variables, as these social determinants are highly interrelated.

### Statistical analyses

All statistical analyses were conducted in SAS 9.4 (SAS Institute, Inc., Cary, North Carolina) and accounted for the complex survey design and non-response. Standard descriptive statistics, including weighted medians and interquartile ranges for continuous variables and weighted percentages for categorical variables, were used to characterise the study population. In order to calculate age- and sex-adjusted mean pathogen burden overall and by PIR, educational attainment, and racial/ethnic categories in each wave of continuous NHANES, we used linear regression models. To assess change in the relative magnitude of pathogen burden disparities over time, we then calculated a ratio measure of the relative burden in each socioeconomic group compared to a referent group. These ratios were calculated by dividing the mean pathogen burden in each group by the mean pathogen burden in the referent group. Trends were plotted graphically and Pearson correlation tests were used to determine any statistically significant linear associations between calendar year and magnitude of disparities.

## Results

Table 1 displays the prevalence of each pathogen in the study population by study wave. Of note, the prevalence of HSV-1 decreased from 62.5% in 1999–2000 to 52.4% in 2013–2014 (*P* < 0.00001), and the prevalence of HSV-2 decreased from 21.2% to 14.3% (*P* < 0.00001). Table 2 displays descriptive statistics for the total study population and stratified by the two socioeconomic indicators of PIR and educational attainment. The lowest socioeconomic groups were younger and had a higher proportion of non-Hispanic Black or Hispanic participants compared to higher socioeconomic groups. Overall, 58.6% of participants were seropositive for HSV-1, 18.8% for HSV-2, 54.2% for CMV, 23.2% for HPV, and 10.9% for *T. gondii.* With the exception of HPV, lower SES was associated with a higher prevalence for each pathogen.

**Table 2.** Descriptive statistics of 1999–2014 NHANES population by socioeconomic indicators, *n* = 17 660

	Overall	PIR category				Educational attainment		
		<1.3	1.3–1.85	>1.85–3.5	>3.5	<HS	HS diploma	>HS
*N*	17 660	5587	2308	4104	5474	4082	9558	4020
Age (years), median (IQR)	35 (27, 42)	31 (24, 39)	32 (25, 40)	34 (27, 41)	37 (30, 44)	34 (26, 41)	34 (26, 42)	36 (29, 42)
Female, %	50.8	55.6	50.6	49.5	48.8	47.1	50.7	53.2
Race, %								
Non-Hispanic White	65.8	48.3	51.6	65.1	80.1	39.4	67.7	77.1
Non-Hispanic Black	11.4	16.9	15.6	12.2	6.7	15.1	12.7	6.8
Mexican American	10.1	18.5	17.2	9.3	3.9	29.4	8.1	3.0
Other Hispanic	6.3	10.5	8.3	6.5	3.2	11.3	6.1	3.6
Other	6.4	5.9	7.3	6.8	6.2	4.7	5.4	9.4
Birth country, %								
U.S.-born	82.1	74.0	73.2	83.7	90.2	62.3	87.3	83.1
Foreign-born	17.9	26.0	26.8	16.3	9.8	37.7	12.7	16.9
Refused/don't know	0.1	0.0	0.0	0.0	0.0	0.0	0.0	0.0
Marital status, %								
Married	53.1	37.0	46.1	53.7	64.2	47.7	49.3	63.8
Widowed	0.5	0.8	0.8	0.4	0.3	0.9	0.5	0.3
Divorced/separated	10.8	14.0	12.3	11.1	8.3	11.2	12.6	7.0
Never married	25.5	33.4	29.1	25.1	20.3	24.8	27.4	22.2
Living with a partner	10.1	14.7	11.7	9.7	6.9	15.4	10.2	6.7
Education level, %								
<HS diploma	16.1	34.5	24.1	13.6	4.8	–	–	–
HS diploma, some college, AA	56.0	57.4	62.3	66.4	47.4	–	–	–
College graduate+	27.9	8.0	13.6	20.0	47.9	–	–	–
Ratio of family income to poverty, %								
<1.30	23.0	–	–	–	–	49.9	23.6	6.6
1.30–1.85	11.1	–	–	–	–	16.8	12.3	5.4
>1.85–3.5	24.7	–	–	–	–	21.0	29.3	17.7
>3.5	41.2	–	–	–	–	12.3	34.8	70.4
HSV-1 (% seropositive)	58.6	68.3	64.5	58.7	51.2	77.5	59.0	53.1
HSV-2 (% seropositive)	18.8	24.9	21.1	19.0	14.4	25.8	20.1	12.2
CMV (% seropositive)	54.2	69.4	61.0	54.9	43.6	76.5	54.3	39.3
HPV (% seropositive)	23.2	25.3	22.5	23.3	21.9	26.6	24.1	19.5
*T. gondii* (% seropositive)	10.9	15.5	15.1	9.8	7.5	21.3	10.3	6.7

Table S1 displays sociodemographic characteristics for the eligible populations by each study year. The proportion of college graduates increased from 26.2% of the population in 1999 to 30.7% in 2014. The proportion of the population in the most impoverished category (PIR < 1.30) increased over the 16 years from 22.0% in 1999–2000 to 26.4% in 2013–14.

**Fig. 1.**
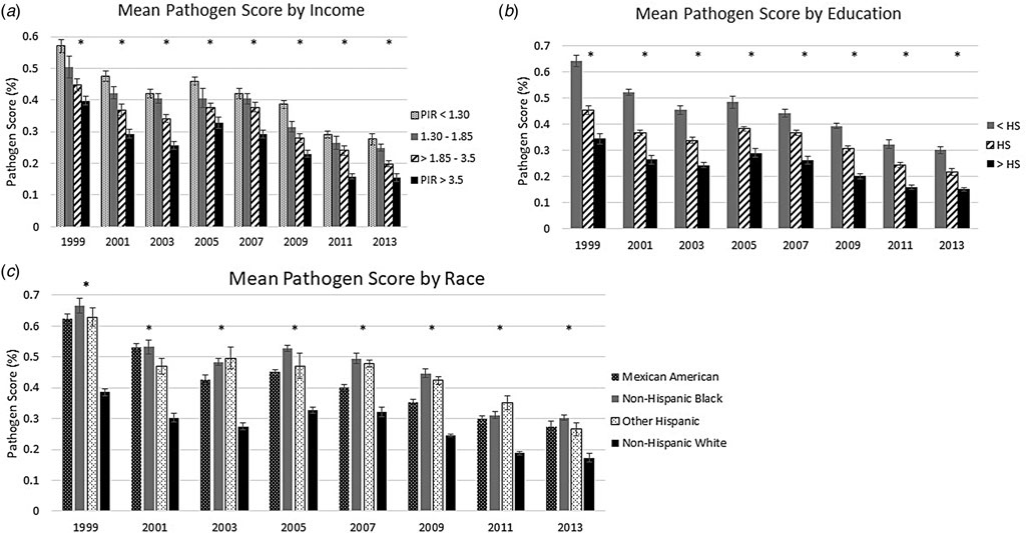
Mean pathogen burden, 1999 to 2014, stratified by socioeconomic and demographic indicators. (a) shows the mean sex- and age-adjusted pathogen burden for each year stratified by the category of PIR; (b) shows the mean sex- and age-adjusted pathogen burden for each year stratified by educational attainment and (c) shows the mean sex- and age-adjusted pathogen burden for each year stratified by the category of racial and ethnic identity. An asterisk (*) above a wave indicates that the exposure was statistically significant compared to the referent group, at *α* = 0.05.

Across all study years, decreasing SES was associated with a higher age- and sex-adjusted mean pathogen burden (see Fig. 1). In 1999–2000, those with a PIR below 1.3 had a mean pathogen burden of 57.0%, while those with a PIR greater than 3.5 had a mean pathogen burden of 39.8%. By the end of the study period, those in the lowest income category had a mean pathogen burden of 27.6% while those in the highest income category had a mean pathogen burden of 15.5%. Educational disparities in pathogen burden were even greater than those observed for household income. In 1999–2000, the mean pathogen burden was 64.1%, for those with less than a high school diploma, 45.6% for those with a high school diploma and 34.4% for those with more than a high school diploma. In the final study years, the mean pathogen burdens were 30.2%, 21.7% and 15.1%, for those with less than a high school diploma, those with a high school diploma and those with more than a high school diploma, respectively.

We also found persistent racial and ethnic disparities in the sex- and age-adjusted mean pathogen burdens across time (Fig. 1). Those identifying as non-Hispanic White had a significantly lower mean pathogen burden across all waves compared to Mexican Americans, Other Hispanics, and non-Hispanic Blacks, with a mean pathogen burden of 38.5% in 1999–2000 compared to above 60% for the other groups. This pattern continued through to 2013–2014, when non-Hispanic Whites had a mean pathogen burden of 17.3%, compared to 27.4% for Mexican Americans, 30.2% for non-Hispanic Blacks, and 26.5% for Other Hispanics.

**Fig. 2.**
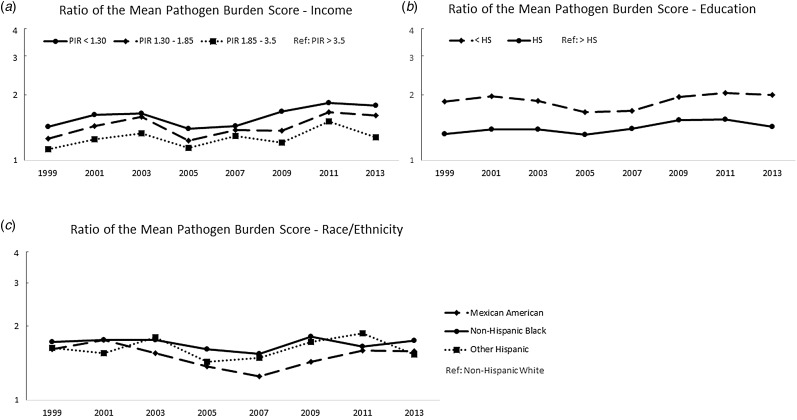
Ratios of mean pathogen burden, 1999 to 2014, by socioeconomic and demographic indicators. (a) shows the ratio of the mean sex- and age-adjusted pathogen burdens for each year with high PIR as the referent group; (b) shows the ratio of the mean sex- and age-adjusted pathogen burdens for each year with high-educational attainment as the referent group and (c) shows the ratio of the mean sex- and age-adjusted pathogen burdens for each year with non-Hispanic Whites as the referent group.

Figure 2 shows the ratios of the mean pathogen burdens, further demonstrating that the relative magnitude of these disparities persisted across the study period. For example, in 1999–2000, those with a PIR < 1.3 had a mean pathogen burden 1.4 times the mean burden of those with a PIR > 3.5 (30%); by 2013–2014, the ratio had increased to 1.8 times the mean burden. Similar trends were observed for education and race/ethnicity. Correlation coefficients, calculated to test linear associations between the ratio measures and calendar year, are shown in Table S2. Consistent with Figure 2, these results did not suggest a reduction in the magnitude of the pathogen burden disparities over time with the exception of a statistically significant trend comparing those with a high school diploma to those with a college degree or higher. This disparity in pathogen burden increased over time (*r* = 0.71 and *P*-value = 0.049).

Figure S2 and Table S3 show the results of our sensitivity analysis that was limited to HSV-1 and HSV-2, which were measured across all study years. Results were similar to those seen with the total pathogen burden score assessed in the primary analysis and suggested that the disparity in pathogen burden by income may even be widening.

## Discussion

In this paper, we assessed temporal trends in socioeconomic and racial/ethnic disparities in pathogen burden over 16 years. Across all 16 years, the highest mean pathogen burden was among those belonging to the lowest SES category. We also found that non-Hispanic Whites consistently had the lowest mean pathogen burden compared to the other racial/ethnic categories. While the overall prevalence of most pathogens declined over the study period, we found no evidence that SES and racial/ethnic disparities in pathogen burden are declining over time. In fact, for educational attainment, the disparities appeared to be widening. To our knowledge, ours is the first study to investigate temporal trends in socioeconomic and racial/ethnic disparities in pathogen burden.

Earlier research has posited several potential pathways linking social disadvantage with a higher prevalence of infections. First, those in socially disadvantaged groups are more likely to be exposed to infections due to factors like overcrowding, and poor neighbourhood environments and public sanitation [32]. Additionally, those in low SES groups might be more susceptible to infection through influences on innate and adaptive immunity, genetic and epigenetic changes, or influences of pre-existing comorbidities that can modify immunity [33, 34].

The higher burden of infection among those in low SES groups is compounded by the fact that these same individuals may be less able to control infections once established. Reasons for this are several fold and may include mechanisms related to modifications in immune response to existing chronic or latent infections, influences on comorbidities, and genetic and epigenetic changes [35, 36]. For example, prior research has documented that low SES is associated with higher levels of CMV antibodies, suggesting a diminished ability to keep the virus in a quiescent state [37]. This is further supported by studies that have found that herpesviruses are indicators of immune system aging and may even play a role in immunosenescence and age-related decline [15]. This may in turn lead to a decreased ability to control infections. A higher burden of latent infections among socially disadvantaged groups, coupled with a potentially diminished capacity to keep infections in a quiescent state, suggests an important role for the immune system in perpetuating disparities in long-term health.

Our study has limitations to note. First, seropositivity does not capture variation in immune response, and future investigations that assess markers such as infection-specific IgG levels would further our understanding of the impact that disparities in pathogen burden may have on health. Additionally, the HPV vaccine may have affected the seroprevalence of HPV in a small subset of our population, particularly in the later years of the study period, which we could not capture in our data. Differential access to HPV and other vaccines may influence trends in pathogen burden disparities and warrants further study as the vaccines become increasingly more common. It is also important to note that NHANES did not consistently measure the same pathogens from year-to-year, which we attempted to account for by standardising the pathogen burden measure by the total number of pathogens tested. The extent to which this variation may have affected our results cannot be assessed. However, the results of our sensitivity analysis are encouraging and showed that using a measure of pathogen burden including only HSV-1 and HSV-2, which were available in all study years, produced trends consistent to those observed in the primary analysis. Our analysis was also limited to the pathogens measured in NHANES and subsequent studies that incorporate additional latent pathogens, such as EBV, may provide additional insights. Finally, self-reported income is often misreported due to social desirability and therefore may be subject to misclassification bias.

Despite these limitations, our study also had a number of strengths, including 16 years of population-based data, a relatively large sample size, and a study sample representative of the non-institutionalised civilian U.S. population.

Previous research has demonstrated socioeconomic and racial and ethnic disparities in pathogen burden among participants in NHANES [10, 11, 26], and our results support and significantly add to this prior work by comparing burden across waves and demonstrating that the disparities in pathogen burden in the U.S. have persisted over 16 years.

Understanding the mechanism through which these disparities arise is essential to reducing and preventing the disproportionate burden of disease carried by socially disadvantaged groups. Our findings that pathogen burden continues to be higher among low SES groups and racial/ethnic minorities may provide tangible points for intervention to mitigate health disparities in the U.S. Further research on measures for preventing persistent infections among the most at-risk groups is warranted. This is particularly relevant given that, as our sensitivity analyses indicate, the disparities may actually be widening despite evidence that prevalence of these infections is decreasing slightly. The higher burden of latent pathogens and potentially diminished immune response among disadvantaged populations constitute a silent epidemic that have allowed these pathogens to continue to fall under the radar of most policy makers and healthcare workers. The Council for State and Territorial Epidemiologists has made some progress in this regard by highlighting efforts to track infection prevalence by area-based (e.g. census tract) socioeconomic indicators as part of the Healthy People 2020 goals [38]. Further research will also be needed on the biological mechanisms by which multiple pathogens influence health and mortality as an important next step for elucidating the utility of interventions for preventing these infections in the U.S.

## Supplementary material

For supplementary material accompanying this paper visit https://doi.org/10.1017/S0950268819001894. click here to view supplementary material
