# Supplementary material for: Persistent socioeconomic and racial and ethnic disparities in pathogen burden in the United States, 1999–2014
Source: Epidemiol Infect. 2019 Nov 11;147:e301. doi: 10.1017/S0950268819001894 (PMC6873154; doi:10.1017/S0950268819001894)
Supplement: Supplementary file 1 [file S0950268819001894sup001.docx]

**Figure S1. Study Inclusion Flowchart by NHANES Wave, 1999-2014.**

* based on infection and covariate data availability

**Figure S2. Sensitivity Analysis: Ratios of Mean HSV-1 + HSV-2 Burden, 1999 to 2014, by socioeconomic and demographic indicators.** Panel A) shows the ratio of the mean sex- and age-adjusted HSV-1 + HSV-2 burdens for each year with high PIR as the referent group; Panel B) shows the ratio of the mean sex- and age-adjusted HSV-1 + HSV-2 burdens for each year with high educational attainment as the referent group; Panel C) shows the ratio of the mean sex- and age-adjusted HSV-1 + HSV-2 burdens for each year with non-Hispanic Whites as the referent group.

| **Table S1. Descriptive Statistics of 1999-2014 NHANES Population by Study Wave, n = 17,660** | | | | | | | | | |
| --- | --- | --- | --- | --- | --- | --- | --- | --- | --- |
|  |  | **1999-2000** | **2001-2002** | **2003-2004** | **2005-2006** | **2007-2008** | **2009-2010** | **2011-2012** | **2013-2014** |
| N |  | 1775 | 2349 | 2097 | 2332 | 2327 | 2484 | 2190 | 2112 |
|  |  |  |  |  |  |  |  |  |  |
| Age (years), median (IQR) |  | 34 (27, 41) | 35 (27, 42) | 35 (27, 42) | 35 (27, 42) | 35 (27, 42) | 34 (27, 42) | 34 (26, 42) | 34 (26, 41) |
|  |  |  |  |  |  |  |  |  |  |
| Female, % |  | 51.4 | 51.1 | 50.0 | 51.0 | 50.9 | 51.4 | 49.9 | 50.8 |
| Race, % |  |  |  |  |  |  |  |  |  |
| *Non-Hispanic White* |  | 68.3 | 67.8 | 67.8 | 67.2 | 66.2 | 64.2 | 62.3 | 61.9 |
| *Non-Hispanic Black* |  | 10.9 | 11.6 | 12.3 | 12.4 | 11.0 | 11.4 | 11.3 | 10.4 |
| *Mexican American* |  | 7.3 | 9.4 | 10.5 | 10.2 | 10.8 | 10.9 | 10.4 | 11.2 |
| *Other Hispanic* |  | 9.1 | 6.3 | 4.3 | 4.1 | 5.9 | 6.4 | 7.4 | 6.9 |
| *Other* |  | 4.3 | 4.9 | 5.0 | 6.2 | 6.0 | 7.1 | 8.5 | 9.6 |
| Birth country, % |  |  |  |  |  |  |  |  |  |
| *US-born* |  | 82.6 | 84.7 | 83.5 | 81.9 | 81.8 | 80.6 | 80.1 | 81.4 |
| *Foreign-born* |  | 17.4 | 15.3 | 16.5 | 18.1 | 18.2 | 19.4 | 19.9 | 18.6 |
| Marital status, % |  |  |  |  |  |  |  |  |  |
| *Married* |  | 54.3 | 56.4 | 54.2 | 55.7 | 52.7 | 50.4 | 49.0 | 51.6 |
| *Widowed* |  | 0.3 | 0.6 | 0.7 | 0.4 | 0.5 | 0.4 | 0.9 | 0.3 |
| *Divorced/Separated* |  | 12.9 | 10.2 | 11.5 | 10.0 | 11.0 | 11.7 | 9.6 | 9.9 |
| *Never married* |  | 26.0 | 24.2 | 24.7 | 21.7 | 25.2 | 26.3 | 29.3 | 27.7 |
| *Living with a partner* |  | 6.6 | 8.7 | 8.9 | 12.2 | 10.7 | 11.2 | 11.2 | 10.5 |
| Education level, % |  |  |  |  |  |  |  |  |  |
| *< HS diploma* |  | 18.3 | 16.1 | 15.0 | 15.9 | 19.1 | 17.1 | 13.7 | 13.0 |
| *HS diploma, some College, AA* | | 55.5 | 58.4 | 60.2 | 56.0 | 54.3 | 54.7 | 52.5 | 56.3 |
| *College Graduate +* |  | 26.2 | 25.4 | 24.8 | 28.2 | 26.6 | 28.1 | 33.8 | 30.7 |
| Ratio of family income to poverty, % |  |  |  |  |  |  |  |  |  |
| *<1.30* |  | 22.0 | 21.6 | 21.6 | 18.1 | 23.4 | 24.7 | 26.9 | 26.4 |
| *1.30-1.85* |  | 11.3 | 11.1 | 11.2 | 9.7 | 10.6 | 13.0 | 12.4 | 9.7 |
| *>1.85-3.5* |  | 23.6 | 25.0 | 26.3 | 27.9 | 23.5 | 24.0 | 21.7 | 25.4 |
| *>3.5* |  | 43.1 | 42.3 | 40.9 | 44.3 | 42.5 | 38.3 | 39.0 | 38.5 |
|  |  |  |  |  |  |  |  |  |  |

| **Table S2. Correlation Coefficients for Study Year and Disparity Measures, Pathogen Burden** | | | |
| --- | --- | --- | --- |
|  | r |  | p-value |
| Ratio of family income to poverty, % |  |  |  |
| *<1.30* | 0.6461 |  | 0.0835 |
| *1.30-1.85* | 0.5520 |  | 0.1561 |
| *>1.85-3.5* | 0.5173 |  | 0.1892 |
| *>3.5* | -ref- |  |  |
|  |  |  |  |
| Education level, % |  |  |  |
| *< HS diploma* | 0.3261 |  | 0.431 |
| *HS diploma, some College, or AA degree* | 0.7087 |  | 0.0491 |
| *College Graduate or above* | -ref- |  |  |
|  |  |  |  |
| Race, % |  |  |  |
| *Non-Hispanic White* | -ref- |  |  |
| *Non-Hispanic Black* | -0.0928 |  | 0.827 |
| *Mexican American* | -0.2862 |  | 0.492 |
| *Other Hispanic* | 0.1364 |  | 0.747 |

| **Table S3. Sensitivity Analysis: Correlation Coefficients for Study Year and Disparity Measures, HSV-1 + HSV-2 Burden** | | | |
| --- | --- | --- | --- |
|  | r |  | p-value |
| Ratio of family income to poverty, % |  |  |  |
| *<1.30* | 0.9056 |  | 0.002 |
| *1.30-1.85* | 0.7906 |  | 0.020 |
| *>1.85-3.5* | 0.7103 |  | 0.048 |
| *>3.5* | -ref- |  |  |
|  |  |  |  |
| Education level, % |  |  |  |
| *< HS diploma* | 0.6059 |  | 0.111 |
| *HS diploma, some College, or AA degree* | 0.6430 |  | 0.085 |
| *College Graduate or above* | -ref- |  |  |
|  |  |  |  |
| Race, % |  |  |  |
| *Non-Hispanic White* | -ref- |  |  |
| *Non-Hispanic Black* | 0.3937 |  | 0.335 |
| *Mexican American* | 0.0854 |  | 0.032 |
| *Other Hispanic* | 0.2536 |  | 0.545 |
